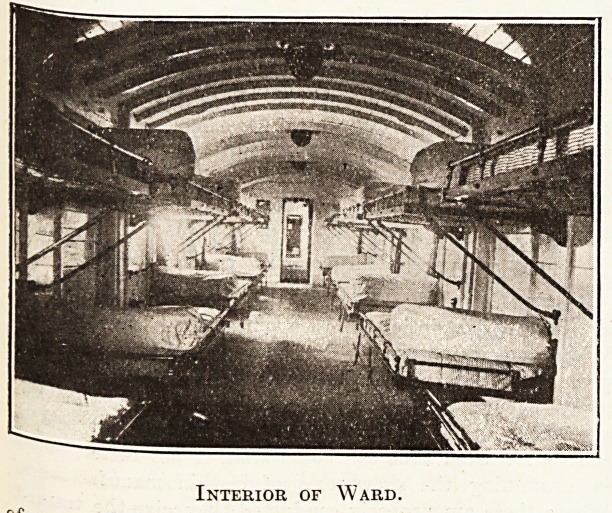# An Irish Hospital Train

**Published:** 1914-10-10

**Authors:** 


### An Irish Hospital Train.

We referred in The Hospital of September 26 to the hospital trains in France, and quoted a writer who pointed out that the chief difficulty concerning them is their limited accommodation. We have also described briefly (The Hospital, September 19, p. 665) the main features of the ambulance train which has been built by the Great Southern and Western Railway Company in Ireland, and as its features become especially interesting in the light of the criticism mentioned above, we publish this week, by the courtesy of Mr. E. A. Watson,

Outside View of Ambulance Train Complete.

locomotive carriage and waggon superintendent to the Great Southern and Western Railway, two illustrations, one of the complete train, and the second of one of the five twenty-bed wards provided by it. The arrangement of the beds in two tiers is interesting, and a point has been made of the corridors, which are constructed sufficiently wide to accommodate a stretcher. The train, which, complete in itself, consists of nine coaches, thus has accommodation for 100 patients and staff.

While it must be remembered that this particular ambulance train was designed to meet the requirements

Interior of Ward.

of wounded who might be landed at the south, south-east, and south-west ports of Ireland, still a train intended for use at the Front itself could not have materially more accommodation for its size. It does not need Dr. J. Scott Riddell, whose "Manual of Ambulance" has many points of interest, to remind us that to be useful such hospital trains, the accommodation of which is so limited, require a clear line, which is almost impossible in the tremendously congested rear of a fighting army. Thus passenger-coaches, or temporary ambulance trains, have to be invoked to take all those wounded capable of sustaining a sitting-up position.

## Figures and Tables

**Figure f1:**
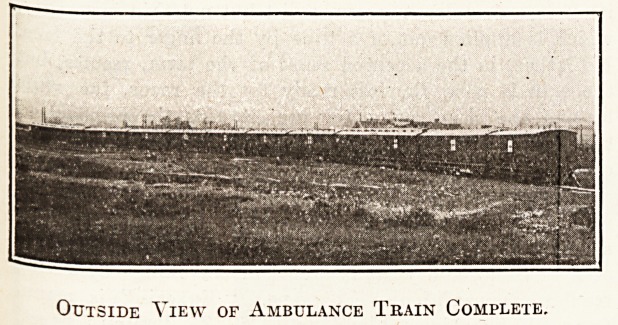


**Figure f2:**